# Irisin prevents dexamethasone-induced atrophy in C2C12 myotubes

**DOI:** 10.1007/s00424-020-02367-4

**Published:** 2020-03-26

**Authors:** Jae Seung Chang, In Deok Kong

**Affiliations:** 1 Department of Physiology, Yonsei University Wonju College of Medicine, 20, Ilsan-ro, Wonju-si, Gangwon-do 26426 South Korea; 2 Yonsei Institute of Sports Science & Exercise Medicine, Wonju, South Korea; 3 Mitohormesis Research Center, Yonsei University Wonju College of Medicine, Wonju, South Korea

**Keywords:** Muscle atrophy, Irisin, Glucocorticoid, FoxO3α, Atrogin-1/MAFbx, MuRF-1

## Abstract

Irisin is a myokine secreted mainly from skeletal muscle that is known for having beneficial metabolic effects via enhancement of energy expenditure and insulin sensitivity. Studies show that irisin also acts as an autocrine/paracrine to promote myogenesis and muscle growth. However, the protective role of irisin against muscular wasting remains unclear. We confirmed that irisin secretion was upregulated by electrical pulse stimulation an in vitro exercise mimetic model. Next, we tested if irisin exerted an anti-atrophic effect on cultured C2C12 myotubes treated with dexamethasone (DEX), a representative inducer of muscular atrophy. Treatment of cultured myotubes with DEX reduced myotube size and increased proteasome activity, which were attenuated by irisin. Also, irisin effectively prevented dephosphorylation of forkhead box O (FoxO) 3α and upregulation of muscle-specific ubiquitin ligases in DEX-treated myotubes. The protective effect of irisin on DEX-mediated myotube atrophy was partially regulated by insulin-like growth factor-1-dependent signaling. These results suggested that irisin may prevent glucocorticoid-induced muscle atrophy by inhibiting FoxO-mediated ubiquitin-proteasome overactivity.

**Electronic supplementary material:**

The online version of this article (10.1007/s00424-020-02367-4) contains supplementary material, which is available to authorized users.

## Introduction

Skeletal muscle atrophy is a debilitating consequence of physiological processes and conditions such as disuse, malnutrition, and aging. It is also a prominent pathological feature of chronic illnesses including cardiac or renal failure, chronic obstructive pulmonary disease, liver cirrhosis, and cancer [6, 11]. Muscle atrophy causes exercise intolerance and an inability to perform daily activity because of muscle weakness and fatigue, which leads to poor quality of life [15, 25]. Excessive loss of muscle mass can exacerbate disease complications due to impaired efficacy of different therapeutic treatments, thus increasing morbidity and mortality [1].

Irisin, a 112-amino acid, hormone-like molecule cleaved from fibronectin type III domain containing 5 (FNDC5) is predominantly secreted from skeletal muscle [20]. Irisin is strongly implicated in muscle growth. Circulating irisin is upregulated by both endurance and strength exercise, and its levels positively correlate with indicators of skeletal muscle mass and levels of insulin-like growth factor-1 (IGF-1), an anabolic hormone of skeletal muscle [10, 13, 18]. Our previous study showed a negative correlation between circulating irisin levels and incidence of pre-sarcopenia and sarcopenia [5]. Correspondingly, mice with null mutations in myostatin, a main negative regulator of muscle growth, display elevated irisin levels and increased muscle growth-related gene expression and skeletal musculature [30]. An increase in irisin secretion has also been observed in vitro, using conditional knockdown of myostatin in C2C12 muscle cells [8]. Recent evidence suggests that irisin exerts its myogenic effects by enhancing the myoblast fusion and protein synthesis pathway [14, 26]. Given the relationship between irisin and skeletal musculature, we questioned if irisin protects against muscular wasting along with being a promyogenic factor.

Dexamethasone (DEX), a synthetic glucocorticoid, is widely used to induce proteolytic muscle atrophy in both in vivo and in vitro models [28]. DEX is commonly used to treat medical conditions such as inflammatory and autoimmune disorders [32]. It also causes a reduction in protein synthesis and promotes the breakdown of proteins related to skeletal muscle mass via the ubiquitin-proteasome system [22, 28]. Because of aggravating catabolic effects concomitant with glucocorticoid treatments, therapeutic approaches that prevent DEX-induced muscle wasting have important clinical implications.

The effect of irisin on glucocorticoid-induced muscle wasting has not yet been established, with studies mostly focused on its myogenic differentiation and regulation of muscle growth-related factors. Therefore, we investigated the hypothesis that irisin, an exercise-responsive myokine, exerts a protective effect against DEX-mediated muscular atrophy in cultured C2C12 myotubes.

## Materials and methods

### Reagents

Dulbecco’s modified Eagles medium, fetal bovine serum, and penicillin/streptomycin were purchased from Hyclone (Logan, UT, USA), and horse serum was from Gibco (Grand Island, NY, USA). Trypsin and TRIzol reagents were purchased from Invitrogen (Carlsbad, CA, USA). DEX and IGF-1 were purchased from Sigma (St Louis, MO, USA), and recombinant irisin (r-irisin) was from Adipogen (Seoul, Korea).

### Cell culture and differentiation induction

C2C12 myoblast cells from the American Type Culture Collection (ATCC, Manassas, VA, USA) were cultivated in Dulbecco’s modified Eagles medium supplemented with 10% fetal bovine serum, 100 U/ml penicillin, and 100 μg/ml streptomycin. For each experimental condition, myoblasts were plated in 100-mm culture dishes (for the measurement of cell diameter), 6-well plates (for western blots), or 96-well plates (for proteasome activity assays). At approximately 90% confluence, 10% fetal bovine serum was replaced with 2% horse serum for 6 days to induce C2C12 myoblast cells to differentiate into myotubes. Cell cultures were maintained in a humidified chamber with 5% CO_2_ at 37 °C, and culture media were changed every other day.

### Chemical and electrical pulse stimulation

As an in vitro exercise model, myotubes were subjected to electrical pulse stimulation (EPS) using a C-dish with carbon electrodes combined with a pulse generator (C-Pace 100; IonOptix, Milton, MA, USA). Either low (1 Hz with 2 ms duration) or high (99 Hz with 1 ms duration, 20 pulses every 20 s) frequency was applied with both EPS modes set to 11.5 V intensity for a cumulative number of pulses for 24 h. Following washing twice with phosphate-buffered saline, EPS treatment was performed under serum-free conditions and conditioned media, and cell lysates were harvested immediately after EPS. For irisin treatment, differentiated C2C12 myotubes were serum starved for 24 h and treated with multiple doses of r-irisin or with an effective dose at different timepoints. To identify anti-atrophic effects of irisin, C2C12 myotubes were subdivided into four groups: (i) control, with cells incubated in serum-free medium containing 100 U/ml penicillin and 100 mg/ml streptomycin; (ii) DEX, with cells treated with 100 μM DEX; (iii) irisin, with cells treated with 100 ng/ml r-irisin; and (iv) DEX + irisin, with cells co-treated with 100 μM DEX and 100 ng/ml r-irisin. All incubations were for 24 or 48 h prior to harvesting cells and performing experiments.

### Western blots

C2C12 myotubes were lysed in RIPA buffer containing protease inhibitor and protein phosphatase inhibitor cocktail. Protein concentrations were determined using a BCA Protein Assay kit (Thermo Fisher Scientific, Waltham, MA, USA). Proteins were separated on 4–12% polyacrylamide gel using sodium dodecyl sulfate-polyacrylamide gel electrophoresis (SDS-PAGE) and transferred to polyvinylidene fluoride membranes. Membranes were blocked in 3 or 5% (w/v) skim milk in Tris-buffered saline with Tween 20 (TBST), followed by incubation overnight at 4 °C with primary antibody against irisin (Adipogen), FNDC5, IGF-1, muscle atrophy F-box (atrogin-1), muscle RING finger-1 (MuRF-1) (Abcam, Cambridge, UK), Ser473-phosphorylated and total Akt, Thr202/Tyr204-phosphorylated and total ERK1/2, Tyr1135-phosphorylated and total IGF-1 receptor (IGF-1R) β, Ser318/321-phosphorylated and total FoxO3α, Thr172-phosphorylated and total AMPKα (Cell Signaling Technology, Danvers, MA, USA), or GAPDH (Santa Cruz Biotechnology, Dallas, Texas, USA). After washing three times for 10 min in TBST, membranes were incubated with peroxidase-conjugated secondary antibody for 1 h followed by washing. Detection of proteins used ECL Western Blotting Substrate (Thermo Fisher Scientific) on ChemiDoc XRS + Systems (Bio-Rad, Hercules, CA, USA). To detect irisin secretion levels, myotubes were incubated for 24 h with serum-free media containing only antibiotics after indicated treatments and washing. Conditioned media were collected and centrifuged at 1000 rpm for 5 min, and supernatants were concentrated using 10-kDa molecular weight cutoff spin filters (Amicon, Millipore, MA, USA). Concentrated culture media samples were loaded onto 15% SDS-PAGE.

### Proteasome activity analysis

A Proteasome-GloTM Chymotrypsin-like Cell-Based Assays kit (Promega, Madison, WI, USA) was used on intact myotubes attached to culture plates according to the manufacturer’s instructions with slight modifications. Myoblasts were seeded at 10,000 cells per well in 100 μl and differentiated in 96-well plates with clear optical bottoms. After 48-h DEX and/or r-irisin treatment of differentiated myotubes, an equal volume of luminogenic substrate specified for chymotrypsin-like protease activity was added to samples. After shaking at 700 rpm using a plate shaker for 2 min and incubation at room temperature for 10 min, luminescence was detected by a luminometer (BioTek Instruments, VT, USA). To confirm assay specificity, the same number of samples was pretreated for 1 h with 10 μM epoxomicin, a proteasome inhibitor. For each sample, proteasome activity was normalized with epoxomicin-pretreated luminescence as the background signal.

### Cell size determination after DEX and/or irisin treatment

After 48-h DEX and/or r-irisin treatment, myotubes were fixed with 4% paraformaldehyde in phosphate-buffered saline. Images were from a Nikon Eclipse TE2000U microscope (Nikon, Avon, MA), captured using Photometrics Cool SNAP CCD camera (Roper Scientific, Tucson, AZ, USA) under phase-contrast microscopy at × 100 magnification. Diameters of individual myotubes were analyzed using MetaMorph 6.1 software (Molecular Devices, Sunnyvale, CA, USA). Average diameters of at least 200 myotubes were determined for each condition at three points separated by 50 μm along the myotube.

### Statistical analysis

All values are presented as mean ± standard error of the mean (SEM) or standard deviation (SD) from at least three separate experiments, and analyses were performed using IBM SPSS Statistics version 23 (IBM Corp., Armonk, NY, USA). *P* values below 0.05 were considered statistically significant. One-way analysis of variance (ANOVA) was used to compare means among multiple groups, followed by Tukey’s post hoc test.

## Results

### Increased irisin expression and secretion in C2C12 myotubes in response to exercise-like conditions

**Fig. 1:**
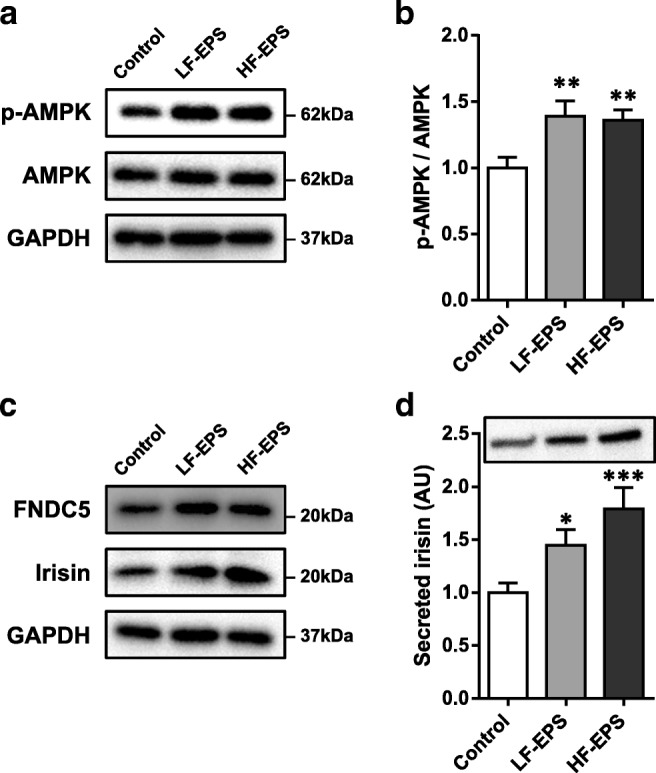
Irisin expression and secretion were upregulated in response to an exercise-like condition in C2C12 myotubes. **A** Representative immunoblot of phosphorylated AMPK (p-AMPK) and total AMPK in C2C12 myotubes treated for 24 h without and with low- or high-frequency mode electrical pulse stimulation (LF-EPS or HF-EPS). **B** Densitometric quantification of relative protein expression of p-AMPK normalized by total AMPK. GAPDH was the loading control. **C** Protein expression and **D** secretion levels of FNDC5/irisin in EPS-treated myotubes. Relative secretion levels in culture medium were normalized to total protein concentration of cell lysates. Bars represent the mean ± SEM of (**B**) or SD of four (**D**) separate experiments. AU, arbitrary unit. *p < 0.05, **p < 0.01, ***p < 0.001 vs. control

To evaluate expression and secretion levels of FNDC5/irisin in response to excitation-contraction coupling, we applied EPS for 24 h to C2C12 myotubes as an in vitro exercise mimetic model. Continuous repetitive contraction and relaxation of myotubes by EPS was observed, and no morphological changes were detected after EPS completion. The exercise-like condition of EPS-induced contraction was confirmed by the ratio of phosphorylated/total AMPK (Fig. 1A and B), which indicated exercise- and contraction-mediated increase in ATP consumption in skeletal muscle cells and tissues. Both low- and high-frequency EPS modalities resulted in increased FNDC5 and irisin protein levels in myotubes (Fig. 1C) and increased irisin secreted into the culture medium (Fig. 1D).

### Irisin activates Akt and ERK1/2 signaling and upregulates IGF-1 expression

**Fig. 2:**
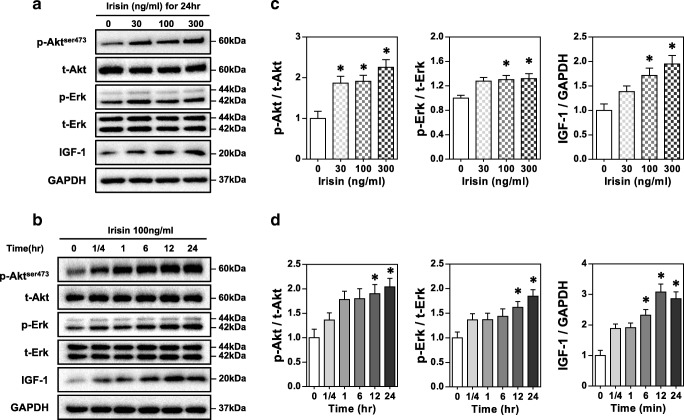
Irisin activates Akt and ERK1/2 signaling and upregulates IGF-1 expression. Representative immunoblot of dose-dependent (**A**) and time-dependent (**B**) effect of irisin treatment on phosphorylation of Akt and ERK1/2 and IGF-1 expression in differentiated C2C12 myotubes. **C** and **D** Densitometric quantification of results in panels **A** and **B**, respectively. GAPDH was the loading control. Bars represent the mean ± SEM of three separate experiments (*n* = 3). *p < 0.05 vs. without irisin treatment

To determine the signaling mediators underlying the myotrophic effects of irisin, we used an in vitro model of r-irisin on C2C12 myotubes starved of serum for 24 h. Western blots showed that 24 h of r-irisin treatment increased IGF-1 protein and phosphorylation of downstream effectors Akt and ERK1/2 in a dose-dependent manner (Fig. 2A and C). Time-dependent increases were also seen at 100 ng/ml r-irisin (Fig. 2B and D). These results indicated that the myotrophic potential of irisin occurred via regulating muscle anabolic factors and downstream signaling.

### Irisin ameliorates atrophic signaling in DEX-induced myotube atrophy

**Fig. 3:**
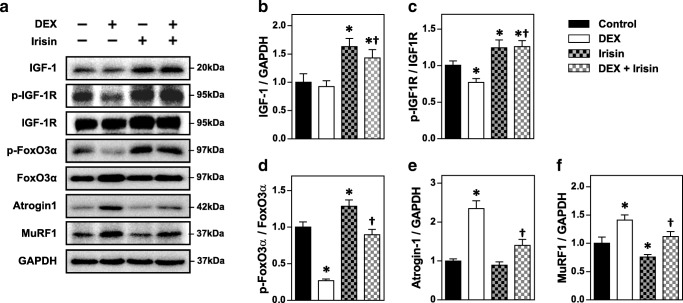
Irisin ameliorates IGF-1 signaling transduction and attenuates FoxO-mediated expression of muscle-specific ubiquitin ligases in dexamethasone-induced myotube atrophy. **A** Effect of irisin treatment on expression of IGF-1, atrogin-1, and MuRF-1 and phosphorylation of IGF-1 receptor (p-IGF-1R) and FoxO3α (p-FoxO3α) in DEX-treated C2C12 myotubes. **B**–**F** Densitometric quantification of protein levels of IGF-1 (**B**), p-IGF-1R (**C**), p-FoxO3α (**D**), atrogin-1 (**E**), and MuRF-1 (**F**). Bars represent the mean ± SEM of three to four separate experiments (*n* = 3–4). DEX, dexamethasone. * *p* < 0.05 vs. control; † *p* < 0.05 vs. DEX

To determine if irisin exerted myogenic effects during atrophic conditions caused by a glucocorticoid, we evaluated expression of IGF-1 and activity of its receptor in DEX-treated myotubes with and without r-irisin. DEX treatment resulted in decreased phosphorylation of IGF-1R, indicating reduced receptor activity. In r-irisin-treated myotubes, DEX had no significant effect on IGF-1 expression or IGF-1R activity (Fig. 3A–C). To investigate the suppressive effect of irisin on DEX-mediated atrophic signaling, we assessed expression of the muscle-specific ubiquitin ligases (atrogenes), atrogin-1 and MuRF-1, and the transcriptional activity of FoxO3α, a critical mediator of atrogenes. DEX treatment increased activation of FoxO3α, as evidenced by decreased phosphorylation (p-FoxO3α) and increased atrogin-1 and MuRF-1 expression. In contrast, r-irisin treatment of myotubes resulted in decreased atrogene signaling and slightly higher FoxO3α phosphorylation and decreased MuRF1 expression. When r-irisin was added to DEX-treated myotubes, the decreased in p-FoxO3α with DEX treatment was abolished, and p-FoxO3α was close to control levels. The increased atrogene expression due to DEX was also significantly lower, suggesting attenuation of ubiquitin-mediated atrophic signaling (Fig. 3A and D–F).

### Irisin attenuates DEX-induced proteolytic activity and myotube atrophy

**Fig. 4:**
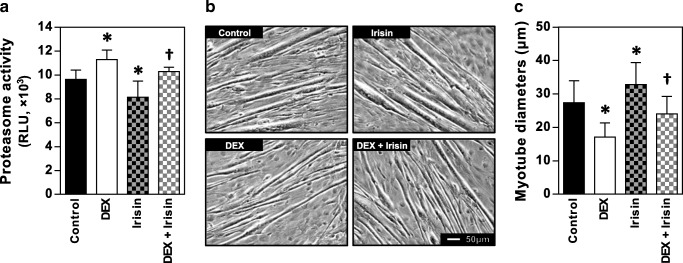
Irisin attenuates dexamethasone-induced proteolytic activity and myotube atrophy. **A** Comparison of the chymotrypsin-like activity of the 26S proteasome determined via cultured cell-based luminescent assay among the four groups. **B** Representative photographs of C2C12 myotubes for control, DEX, irisin, and DEX + irisin treatments. **C** Comparison of myotube diameters among the four groups. Bars represent the mean ± SD of three (**A**, 4 wells per group in each experiment) or four (**C**, 50 myotubes in each experiment) separate cultures. RLU, relative light units. DEX, dexamethasone. * *p* < 0.05 vs. control; † *p* < 0.05 vs. DEX

Next, we investigated if attenuation of muscle atrophy- and hypertrophy-related signaling by r-irisin prevented muscular wasting. We measured the chymotrypsin-like activity of 26S proteasome, considered to be representative of the proteasome proteolytic capacity. Consistent with the molecular alterations, r-irisin treatment reduced baseline proteasome activity. It also attenuated the elevated proteasome activity with DEX in C2C12 myotubes (Fig. 4A). Finally, to confirm the effectiveness of irisin on preserving myotube size, morphological differences were observed among experimental groups of control or treated with DEX and/or r-irisin. Representative photos of myotubes with corresponding treatments are in Fig. 4B. Compared to the control group, exposure to 100 uM DEX for 24 h reduced myotube diameter by 37.4%, whereas treatment with 100 ng/ml r-irisin increased myotube diameters by 19.9%. Myotube diameter was largely restored in the DEX + r-irisin group to 87.8% of control values (Fig. 4C). These results showed that irisin treatment prevented myotube wasting and suggested that this effect correlated with an ubiquitin-proteasome proteolytic pathway.

## Discussion

Controversy about the existence of human irisin was resolved after circulating human irisin was quantitatively confirmed in sedentary and trained individuals by mass spectrometry assays [16]. Reports indicate that irisin positively affects myogenesis by enhancing intramuscular anabolic signaling and protects against unloading- or denervation-induced muscle wasting [7, 26]. However, the endocrine/paracrine effect of this myokine on glucocorticoid-induced myopathy is still unknown. We reasoned that irisin alleviated glucocorticoid-mediated muscular atrophy, since it is a myogenic and exercise-induced factor. To our knowledge, this is the first evidence that irisin has an anti-atrophic potential on a myopathy model caused by a glucocorticoid.

EPS-treated myotubes are confirmed to be closely comparable to muscles of trained mice in an exercise-activated signaling pathway, mitochondrial biogenesis, and substrate metabolism [3, 23]. Emerging evidence indicates that EPS augments the expression and secretion of several myokines in cultured myotubes [23], but whether that was true for irisin was unclear. Only two studies used in vitro models to study irisin, using pharmacological compounds to mimic acute exercise responses [10]. In those studies, despite the increase in PGC-1α mRNA, irisin production remained unaltered or even decreased [17, 27]. By contrast, EPS-evoked myotube contraction in this study resulted in increased irisin expression and secretion. These discrepancies among study results may be attributed to variation in the ability of different approaches to trigger exercise-activated signaling and adaptive responses. Repetitive mechanical stress and intracellular calcium transients do not occur in pharmacological simulation of exercise, which may be crucial factors for irisin production.

The results of this study were consistent with studies that showed irisin stimulates phosphorylation of Akt and ERK1/2 in murine muscle cell and tissue [26, 33]. These molecules are considered important intracellular signaling molecules of IGF-1-mediated muscle growth and hypertrophy [12]. These results, along with increased IGF-1 expression, suggest that irisin leads to stimulation of muscle protein anabolism in an IGF-1-dependent manner [31].

High levels of glucocorticoid in pathological and pharmacological conditions can cause muscle breakdown due to the increased rate of protein degradation, which results from reduced PI3K-Akt signaling and consequent increase in FoxO-mediated proteolysis [6, 9]. Among the three FoxO isoforms in skeletal muscle, the activation of FoxO3 through dephosphorylation is sufficient to promote two crucial ubiquitin ligases, atrogin-1 and MuRF-1. This event leads to the hyperactivity of the proteolytic system [4, 28]. Our results showed that irisin prevented the phosphorylation of FoxO3α and suppressed the expression of atrogenes during normal conditions as well as during DEX treatment. Irisin also attenuated chymotrypsin-like enzyme activity, the major proteolytic activity of 26S proteasome, that serves as the principal machine for regulated protein degradation in eukaryotic cells [19]. Interestingly, reduced functional activity of the proteasome by irisin was observed even in basal condition without DEX treatment. Overall, these results implied that the reduced activities of FoxO subsequently down-regulated ubiquitin ligases. This muscle-specific signaling pathway was at least partly attributable to enhanced IGF-1 signaling through irisin treatment [21]. Finally, we confirmed that the decrease in myotube diameter induced by DEX was prevented by co-treating with irisin.

Although this study was conducted in an in vitro system, previous studies show that DEX-mediated changes in protein turnover rates are quite similar in cultured myotubes and in vivo muscle tissues [29]. We therefore assume that our results predict a preventive effect of irisin on glucocorticoid-induced muscle atrophy in vivo. Further research is needed, however, to understand the exact mechanism of irisin-mediated effects. Identifying the irisin receptors will help in understanding the signaling mechanism which results in the elevation of IGF-1 and its related intracellular signaling. Consequently, this study also suggests that the enhancement of muscle anabolic signaling may also be associated with the mechanisms reported in the recent works where irisin was identified as a novel myokine that improves the glucose dysregulation as well as abnormal lipid metabolism [2, 24].

Taken together, these findings suggest that irisin exerts a protective effect against muscle wasting by counteracting the effect of DEX on FoxO-mediated ubiquitin-proteasome overactivity and restoration of muscular atrophy through IGF-1-mediated signaling. We believe this study provides the basis for understanding the beneficial effects of exercise or pharmacological approaches using myokines to manage glucocorticoid-induced muscle wasting.

## Electronic supplementary material

ESM 1 (PDF 4767 kb)

## Abbreviations

FNDC5: Fibronectin type III domain containing 5
AKT: Protein kinase B
ERK: Extracellular signal-regulated kinase
IGF-1: Insulin-like growth factor-1
DEX: Dexamethasone
FoxO: Forkhead box O
Atrogin-1: Muscle atrophy F-box protein
MuRF-1: Muscle RING-finger protein-1
