# Supplementary material for: Irisin prevents dexamethasone-induced atrophy in C2C12 myotubes
Source: Pflugers Arch. 2020 Mar 26;472(4):495–502. doi: 10.1007/s00424-020-02367-4 (PMC7165150; doi:10.1007/s00424-020-02367-4)
Supplement: Supplementary file 1 — (PDF 4767 kb) [file 424_2020_2367_MOESM1_ESM.pdf]

Supplementary information

Article title: Irisin prevents dexamethasone-induced atrophy in C2C12 myotubes

Journal: *Pflügers Archiv - European Journal of Physiology*

Author: Jae Seung Chang, Ph.D. and In Deok Kong, M.D., Ph.D.

Corresponding author: In Deok Kong, Yonsei University Wonju College of Medicine. E-mail: kong@yonsei.ac.kr

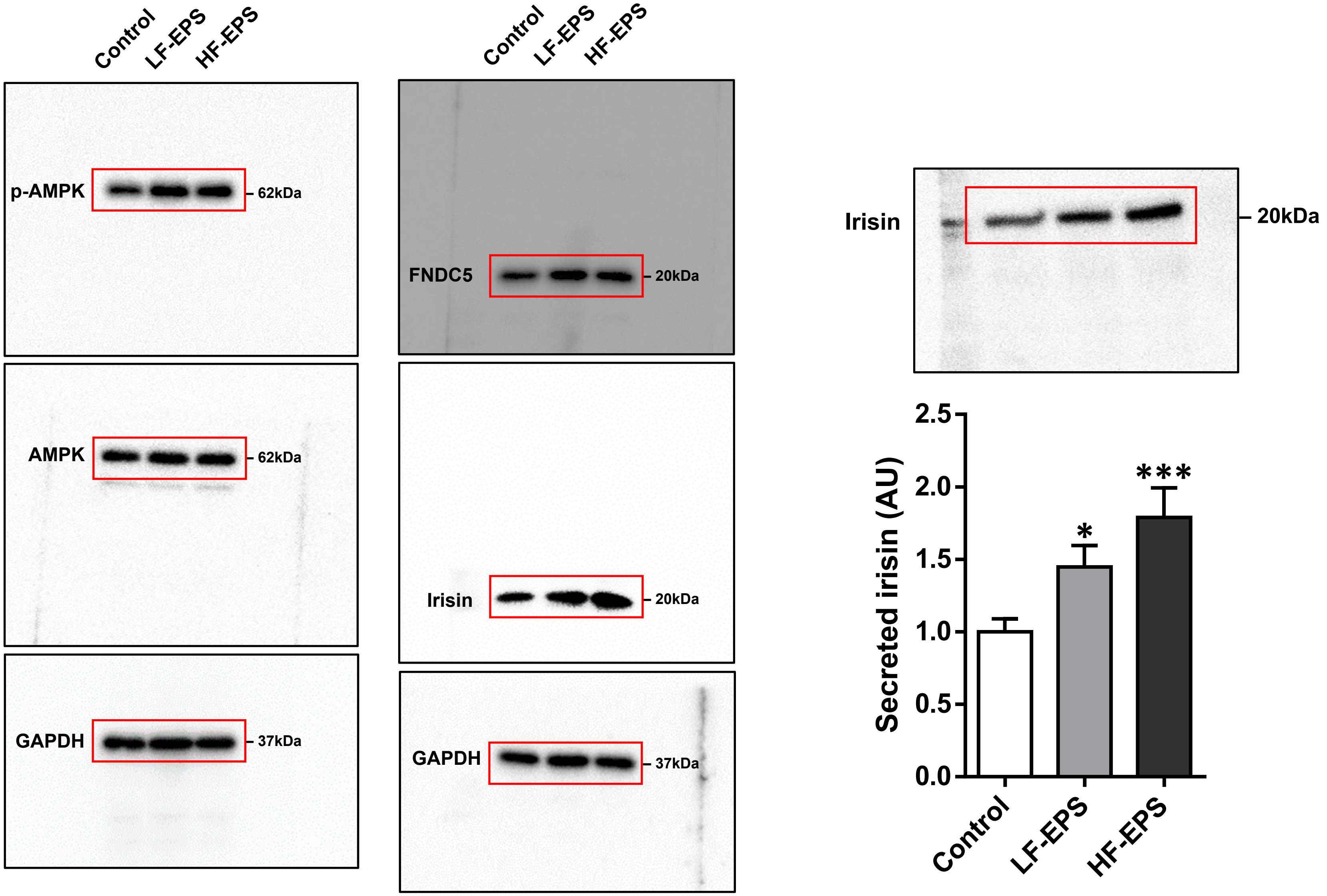

Supplementary Figure 1. Uncropped western blot images shown in Figure 1. Red boxes highlight the cropped segment presented in the main figures.

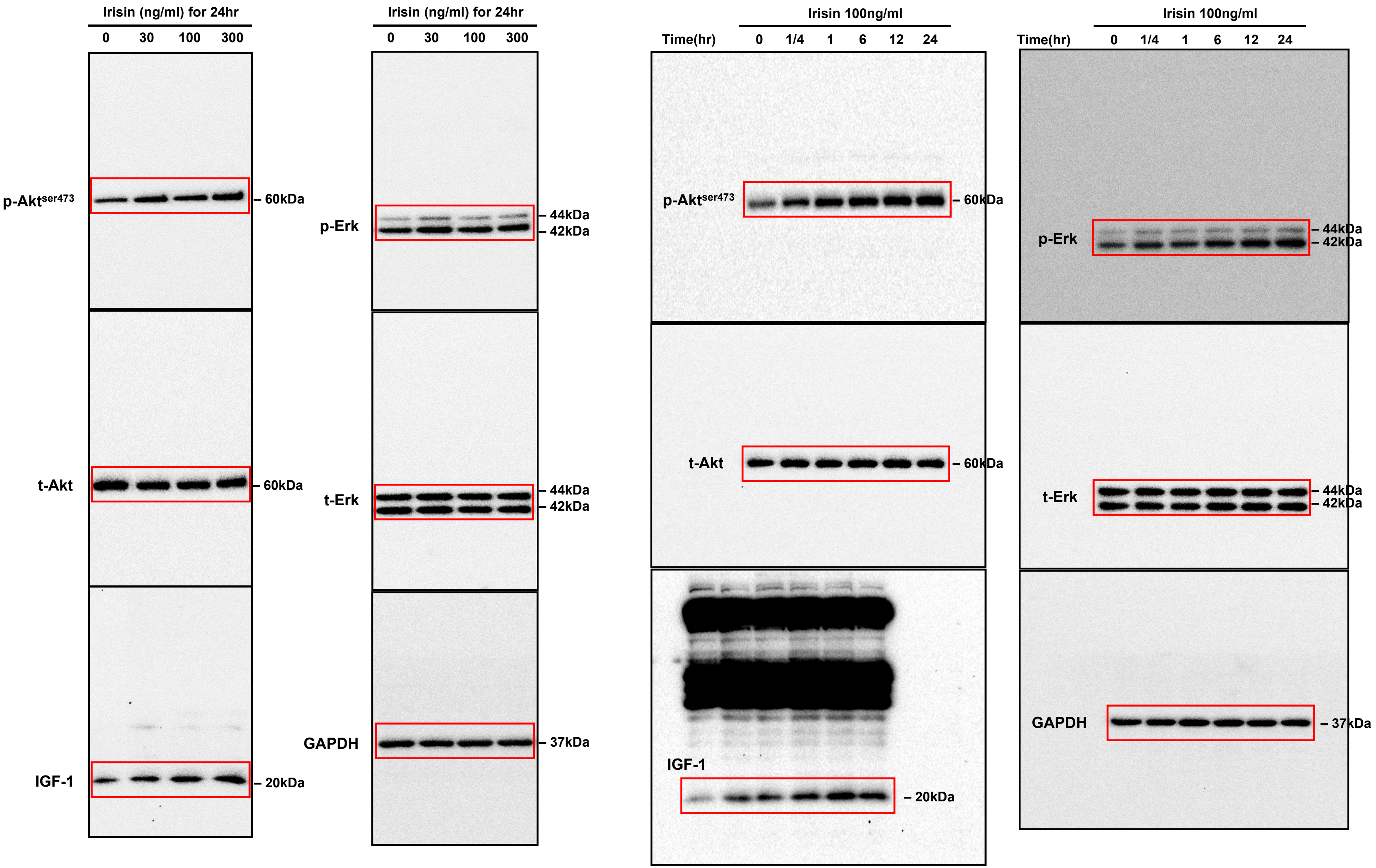

Supplementary Figure 2. Uncropped western blot images shown in Figure 2. Red boxes highlight the cropped segment presented in the main figures.

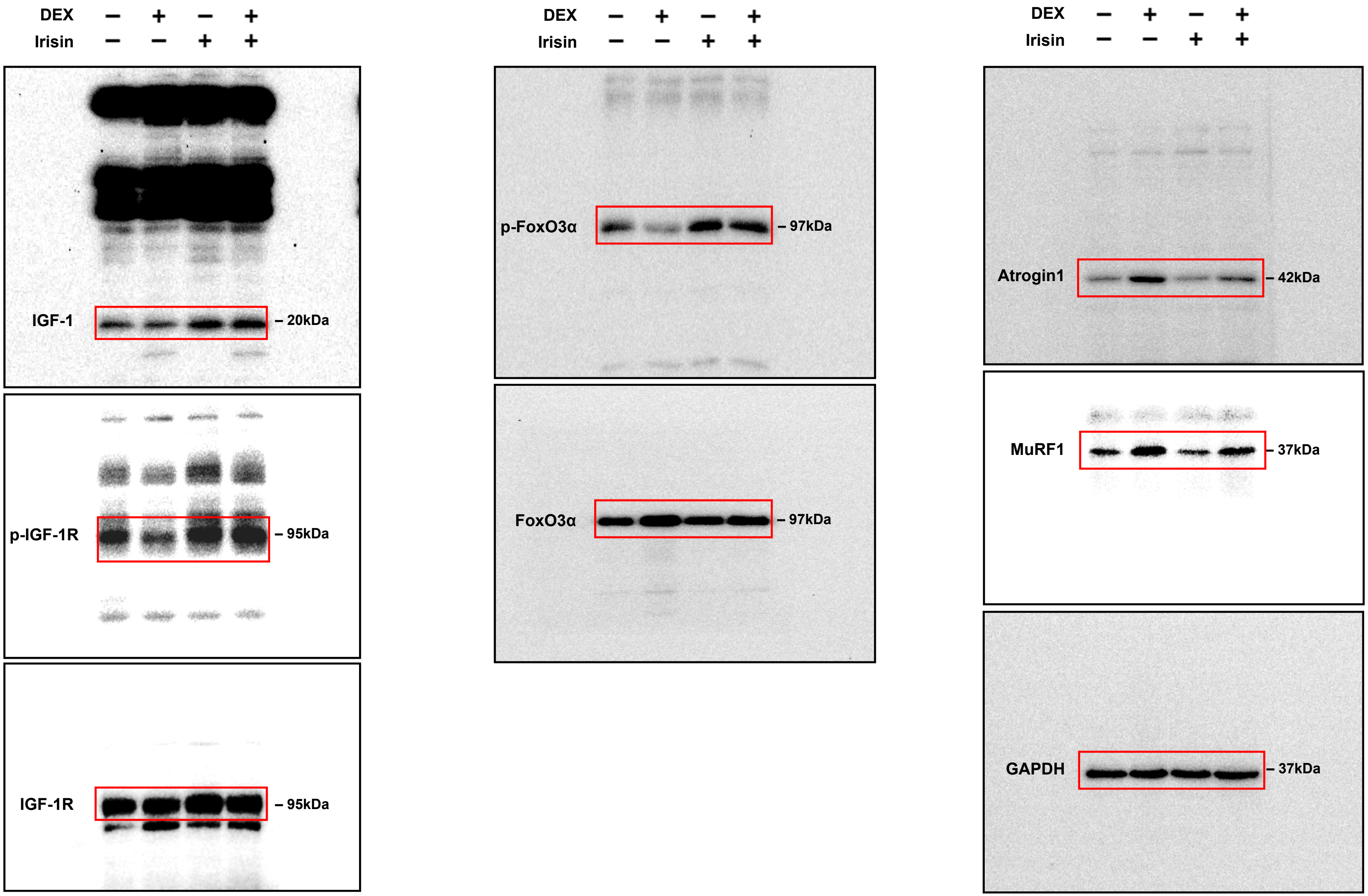

Supplementary Figure 3. Uncropped western blot images shown in Figure 3. Red boxes highlight the cropped segment presented in the main figures.

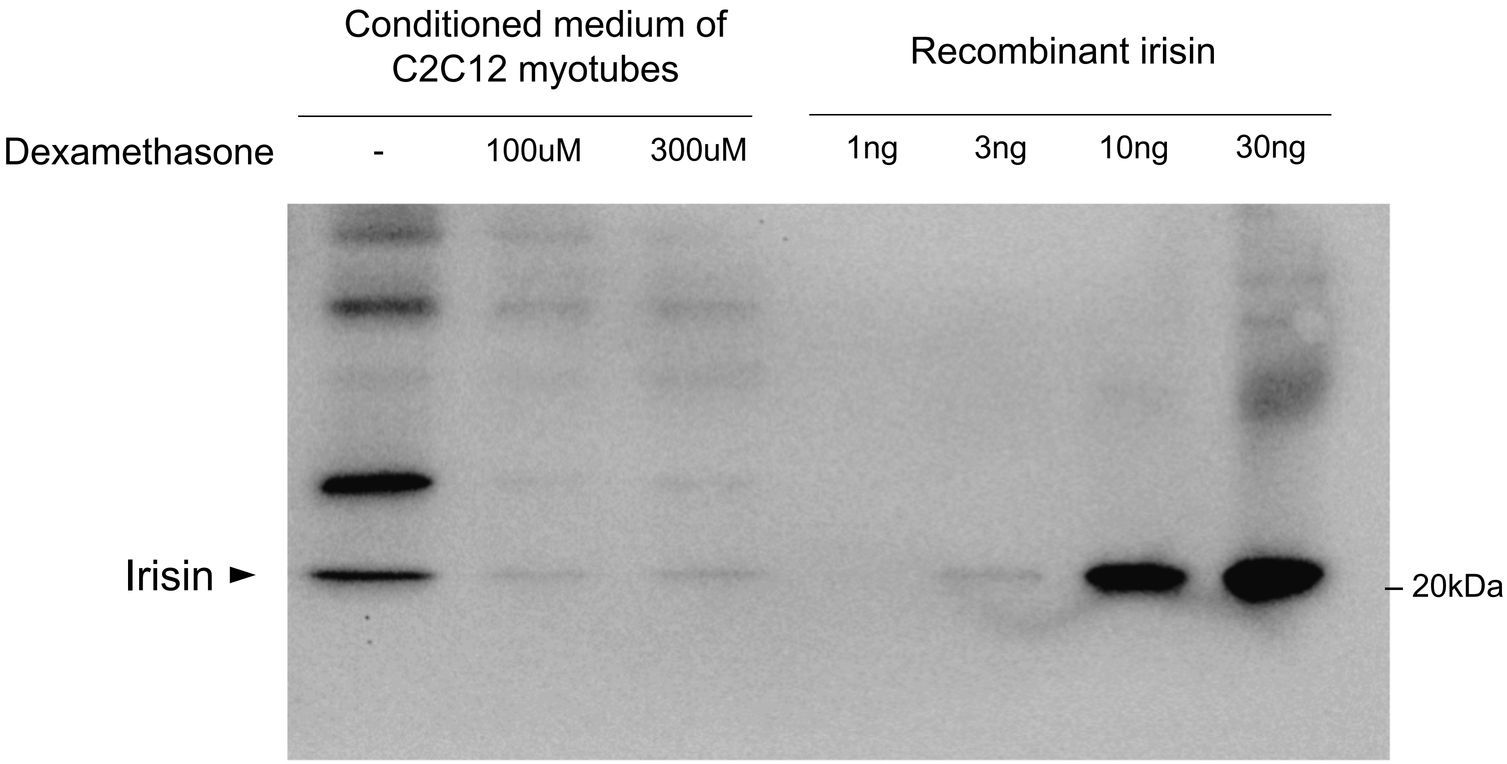

Supplementary Figure 4. A representative image of a quantitative western blot assay used to measure conditioned medium levels of irisin from C2C12 myotubes. Conditioned medium from C2C12 myotubes with or without dexamethasone treatment for 48 hours and multiple doses of recombinant irisin proteins were resolved by SDS-PAGE, transferred to polyvinylidene fluoride membranes, and probed with an antibody against the conserved amine-terminus of irisin by standard Western blotting procedures. The primary antibody was added at a dilution of 1:1000, followed by incubation and washing with an HRP-labeled secondary antibody used at dilution 1:5000. Following removal of second antibody solution, the membrane was washed and exposed to the chemiluminescent enzyme substrate, and signals were captured, digitized, and analyzed using a ChemiDoc XRS+ Imaging System (Bio-Rad, Hercules, CA, USA).
